# Added Value of Computed Tomography to Ultrasonography for Assessing LN Metastasis in Preoperative Patients with Thyroid Cancer: Node-by-Node Correlation

**DOI:** 10.3390/cancers12051190

**Published:** 2020-05-08

**Authors:** Roh-Eul Yoo, Ji-hoon Kim, Inpyeong Hwang, Koung Mi Kang, Tae Jin Yun, Seung Hong Choi, Chul-Ho Sohn, Sun-Won Park

**Affiliations:** 1 Department of Radiology, Seoul National University Hospital, 101 Daehak-ro, Jongno-gu, Seoul 03080, Korea; roheul7@gmail.com (R.-E.Y.); mit3000kr@gmail.com (I.H.); we3001@gmail.com (K.M.K.); radiologyyun@gmail.com (T.J.Y.); verocay1@snu.ac.kr (S.H.C.); neurorad63@gmail.com (C.-H.S.); 2 Department of Radiology, Seoul National University College of Medicine, 103 Daehak-ro, Jongno-gu, Seoul 03080, Korea; swpark8802@gmail.com; 3 Department of Radiology, Seoul Metropolitan Government Seoul National University Boramae Medical Center, 5-Gil 20, Boramae-Road, Dongjak-gu, Seoul 156-707, Korea

**Keywords:** CT, lymph node metastasis, node-by-node correlation, thyroid cancer, ultrasonography

## Abstract

Diagnostic accuracy of US in the evaluation of lymph node (LN) metastasis for thyroid cancer patients is limited. We investigated the value of CT added to US for characterizing LNs in preoperative thyroid cancer patients by node-by-node correlation. A total of 225 primary thyroid cancer patients who underwent LN biopsy were included. Based on node-by-node correlation, 274 LNs were classified into probably benign, indeterminate, and suspicious categories on US, CT, and combined US/CT. Malignancy risks were calculated for each category and were compared between US/CT concordant and discordant cases. On US, CT, and combined US/CT, malignancy risks were 1.7%, 8.7%, and 0% in the probably benign category, 22.4%, 5.9%, and 8.0% in the indeterminate category, and 77.2%, 82.0%, and 75.6% in the suspicious category, respectively. Malignancy risk of the concordant suspicious category was higher than that of the discordant suspicious category (84.7% vs. 43.2%, *p* < 0.001). The addition of CT helped correctly detect additional metastasis in 16.4% of the US indeterminate LNs and in 1.7% of the US probably benign LNs. CT may complement US for LN characterization in thyroid cancer patients by suggesting the diagnostic confidence level for the suspicious category and helping correctly detect metastasis in US indeterminate LNs.

## 1. Introduction

Despite the low mortality rate of papillary carcinoma (PTC) patients, the rates of cervical lymph node (LN) metastases at initial presentation and the time of recurrence have been reported to be relatively high, and thus accurate preoperative imaging diagnosis of LN metastasis has been considered key to reducing the chance of repetitive surgery and operation-related morbidity [1,2,3,4,5,6].

Owing to its wide availability and high-resolution as well as the lack of exposure to ionizing radiation, ultrasonography (US) has been established as the mainstay in the diagnostic imaging work-up during the preoperative evaluation of LN metastasis according to various international guidelines [1,7,8,9,10,11,12]. However, many studies have reported that the US does not have sufficient accuracy for the diagnosis of LN metastasis [13,14,15,16]. Among various factors, the frequent manifestation of US indeterminate LNs that belong to neither a US probably benign nor a suspicious category is regarded as one of the important causes for the limited accuracy of US [8,10,17,18,19,20].

Recently, several articles have reported that the combination of US and CT (computed tomography) has a significantly higher sensitivity than the US alone without a significant difference in specificity, thus supporting the complementary role of CT in the preoperative LN evaluation [21,22,23,24]. However, all of the previous studies were performed on the basis of level-by-level analysis, and the reason why the combination of US and CT had a higher sensitivity and accuracy was not clear, other than the ability of CT to detect additional LN metastasis in US blind spots (e.g., the mediastinum or retropharyngeal area).

Until now, no studies to date have evaluated the value of CT added to US on the basis of node-by-node correlation, and thus the exact incremental role of CT in the characterization of LNs remains elusive. The purpose of this study was to investigate the value of CT added to US for characterizing LNs in preoperative thyroid cancer patients by node-by-node correlation.

## 2. Results

### 2.1. Baseline Characteristics

Of the 274 LNs, the final diagnosis was benign in 143 (52.2%) LNs and metastatic in 131 (47.8%) LNs. Histology of primary tumor comprised PTC (97.1% [266/274]), medullary thyroid carcinoma (1.5% [4/274]), anaplastic carcinoma (0.7% [2/274]), minimally invasive follicular thyroid carcinoma (0.4% [1/274]), and poorly differentiated carcinoma (0.4% [1/274]). The mean largest diameter of the primary tumor was 11.8 mm (range, 2.0−52.0 mm). The mean SDs and LDs were 6.1 mm (range, 2.2−29.1 mm) and 10.7 mm (range, 3.5−49.7 mm) for all LNs, respectively.

### 2.2. Incidences and Malignancy Risks of the US, CT, and Combined US/CT Categories

The incidences of each category of US-, CT-, and combined US/CT-based diagnoses are listed in Table 1. On CT, the incidence of the probably benign category significantly decreased (*p* < 0.001) while that of the indeterminate category significantly increased (*p* = 0.002), as compared with US. This was because many cases (38/58) of the US probably benign LNs were changed to the CT indeterminate LNs. There was no significant difference in the incidence of each category between US- and combined US/CT-based diagnoses (all *p* > 0.05).

Table 2 shows the malignancy risks for the US, CT, and combined US/CT categories. There was no significant difference in malignancy risks between the probably benign and indeterminate categories on CT (*p* = 0.641). Otherwise, the malignancy risks were higher in higher categories (probably benign, indeterminate, and suspicious, in increasing order) for US-, CT-, and combined US/CT-based diagnoses. For the indeterminate category, the malignancy risk was significantly lower on CT (*p* = 0.003) and combined US/CT (*p* = 0.046) than on US.

### 2.3. Malignancy Risks of the Combined US/CT Categories According to the US and CT Categories

Table 3 shows the malignancy risks of the combined US/CT categories according to the US and CT categories. According to the CT categories, the malignancy risks in the same US categories were broad in range (probably benign: 0–25.0%; indeterminate: 0–73.3%; and suspicious: 15.4–84.7%).

**Figure 1:**
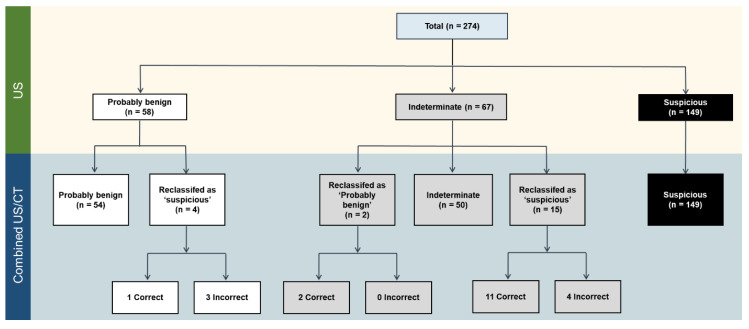
Changes in the LN classification with the addition of CT. CT—computed tomography, LN—lymph node, US—ultrasonography.

**Figure 2:**
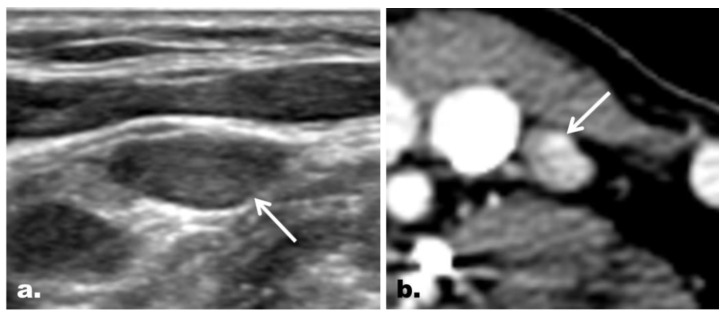
Representative case of correct reclassification of a US indeterminate LN with the addition of CT in a 48-year-old woman with PTC. (**a**) On US, an ovoid LN (arrow) with neither echogenic hilum nor suspicious feature was noted at the left neck level IV. The LN was classified as an indeterminate LN on US. (**b**) The LN, however, was shown to have a focal strong contrast enhancement (arrow) on CT, which led to correct reclassification of the LN as a suspicious LN. The final diagnosis based on FNA turned out to be metastasis. Correct changes in the classification were made in 5.1% (14 of 274) of the LNs enrolled, and the addition of CT helped identify additional metastasis in 6.2% (14 of 225) of the patients enrolled.

A flowchart showing changes in the LN classification with the addition of CT is provided in Figure 1. With regard to the US probably benign cases, 54 of 58 (93.1%) LNs remained as probably benign LNs on combined US/CT. With the addition of CT, 6.9% (4 of 58) of the US probably benign LNs were reclassified as suspicious LNs. Of the four changes in classification, one case (25%) was correct, and three cases (75%) were incorrect. With regard to the US indeterminate cases, 50 of 67 (74.6%) LNs remained as indeterminate LNs on combined US/CT. With the addition of CT, 3.0% (2/67) of the US indeterminate LNs were reclassified as probably benign LNs, while 22.4% (15/67) of the LNs were reclassified as suspicious LNs. Imaging–pathology correlation confirmed that 76.5% (13/17) of the total changes were correct (100.0% [2/2] of cases reclassified as probably benign LNs; 73.3% [11/15] of cases reclassified as suspicious LNs [Figure 2]). The addition of CT helped identify additional metastasis in 1.7% (1/58) of the US probably benign LNs and 16.4% (11/67) of the US indeterminate LNs.

### 2.4. Concordance between the US and CT Categories

**Figure 3:**
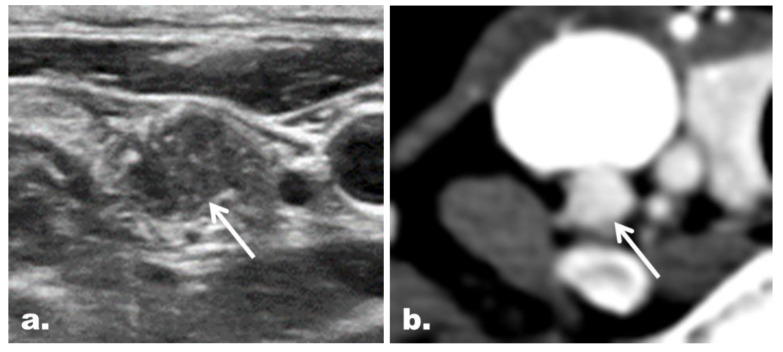
Representative case of a concordant suspicious LN with the final diagnosis of metastasis in a 41-year-old woman with PTC. (**a**) The gray scale US image depicts a round LN (arrow) with microcalcifications at the right neck level IV. The LN was classified as a suspicious LN on US. (**b**) The LN (arrow) showed diffuse strong enhancement on CT and thus was also classified as a suspicious LN on CT.

**Figure 4:**
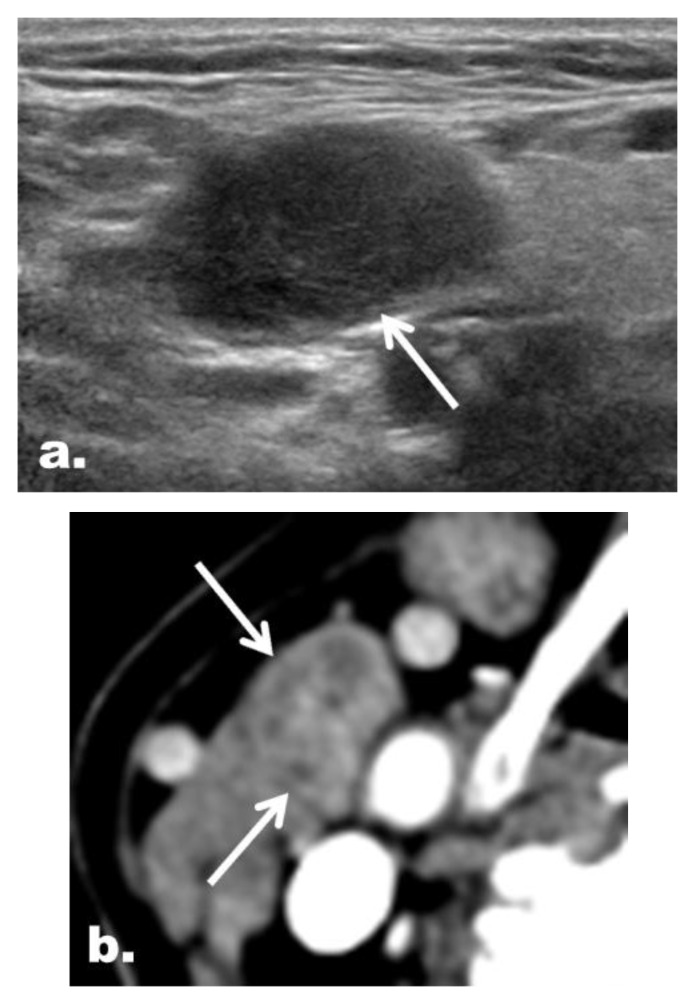
Representative case of a discordant suspicious LN with the final diagnosis of a benign LN in a 61-year-old woman with PTC. (**a**) On US, an ovoid LN (arrow) at the right neck level II had neither echogenic hilum nor suspicious feature and therefore was classified as an indeterminate LN. (**b**) On CT, LN (arrows) was interpreted to have a heterogeneous enhancement and was classified as a suspicious LN.

**Figure 5:**
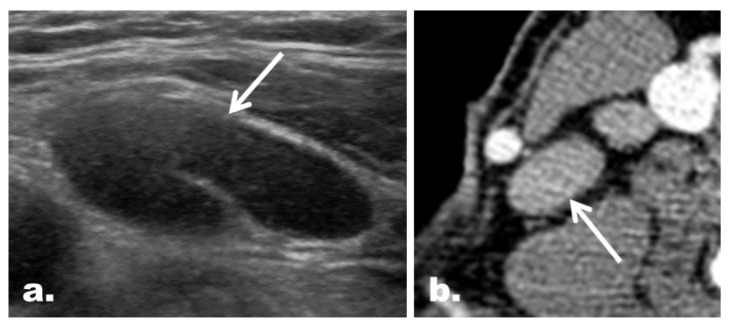
Discordant benign case with the final diagnosis of a benign LN in a 67-year-old woman with PTC. (**a**) On US, an ovoid LN (arrow) with echogenic hilum was found at the right neck level IV. The LN was classified as probably benign LN on US. (**b**) On CT, the LN (arrow) was classified as an indeterminate LN because it showed neither central hilar fat nor central hilar vessel enhancement in the absence of any suspicious feature.

Table 4 summarizes the concordance between the US and CT categories. The US and CT categories were concordant in 197 of 274 LNs (71.9%) and discordant in 77 of 274 LNs (28.1%). The malignancy risk of concordant suspicious cases (Figure 3) was significantly higher than that of discordant suspicious cases (Figure 4) (84.7% [111/131] vs. 43.2% [16/37], *p* < 0.001). On the other hand, the malignancy risk was 0% for benign cases irrespective of concordance (Figure 5).

### 2.5. CT Imaging Features in the Reclassified Cases

Of the 12 LNs that were correctly reclassified as suspicious LNs, focal or diffuse strong enhancement was noted in 11 LNs, and heterogeneous enhancement was noted in one LN. Among them, one LN with focal strong enhancement also showed cystic change. Of the seven LNs that were incorrectly reclassified as suspicious LNs (false positive cases), four LNs were interpreted as having heterogeneous enhancement and three LNs as having strong enhancement. Among the LNs with heterogeneous enhancement, one LN was interpreted as having cystic change. For the two indeterminate LNs that were correctly reclassified as probably benign LNs, hilar vessel enhancement was observed on CT.

### 2.6. Interobserver Aagreement for the US and CT Classification of LNs

Cohen’s unweighted κ coefficients for the US and CT classification of LNs were 0.841 (95% confidence interval [CI]: 0.788, 0.894) and 0.875 (95% CI: 0.822, 0.928), indicating almost perfect agreement for both classifications.

## 3. Discussion

This is the first node-by-node correlation study between CT and US in thyroid cancer patients to investigate the hypothesis that CT has added value to US by helping to characterize LNs detected on US. We found that the addition of CT to US resulted in additional correct characterization of LN metastasis in 5.1% of the total LNs and in 6.2% of the total patients enrolled. In particular, the addition of CT led to correct changes in the classification in 19.4% of the indeterminate LNs, which were not infrequently found in this study population (22.4%), with identification of additional metastasis in 16.4%. The malignancy risk of concordant suspicious cases was significantly higher than that of discordant suspicious cases.

LN metastasis in patients with thyroid cancer may be detected at the time of initial diagnosis or postoperative screening for tumor recurrence, and US imaging is broadly accepted as the first-line imaging modality worldwide for the evaluation of LN metastasis [25,26]. There is consensus among various international guidelines that US findings such as microcalcification, cystic change, hyperechogenicity, or increased vascularization (peripherally or diffusely) are highly specific to malignancy [8,10,12]. On the other hand, an oval shape with a hyperechoic central stripe or vascular flow (representing the preserved fatty hilum) in the absence of any of the aforementioned suspicious features is considered to be specific for benign LNs [8,10,12]. Nonetheless, previous studies have shown that sensitivities of the suspicious findings for the prediction of LN metastasis are limited [13,14,15,16,26]. Moreover, identification of the central fatty hilum may also be challenging in some pathologically benign cases [16,26]. In both situations, LNs end up belonging to neither category, and such LNs in the “gray zone” have been classified as a separate category (i.e., ‘indeterminate’) in several papers and some guidelines [8,10,17,18,19]. Therefore, in routine practice, one would expect that the diagnostic performance for the detection of LN metastasis may be enhanced if US indeterminate LNs can be correctly reclassified into either probably benign or suspicious LNs. In this regard, our results highlighted the added value of CT to US for classifying US indeterminate LNs in the preoperative LN assessment in thyroid cancer patients.

Meanwhile, CT, which is the imaging modality of choice for LN evaluation in other head and neck cancers, remains an adjunct to US and is reserved for selected indications in thyroid cancer patients—for example, bulky nodal disease or suspicious involvement of the mediastinum or deep structures of the neck [12]. However, as in the results from a previous meta-analysis based on nine studies [27], many papers have reported that combination of US and CT results in a significantly higher sensitivity and a similar specificity, as compared with US alone [21,22,23,24]. Concurrently, the clinical benefit of CT over US has been validated, and the role of CT has been advocated in low risk cancer, even in papillary thyroid microcarcinoma [21,28]. In this regard, our study supports the rationale of adding CT to US, demonstrating how and how much CT can be helpful for LN characterization in addition to the detection of additional LN metastasis in US blind spots (e.g., the mediastinum or retropharyngeal area) [23]. Specifically, the analysis of CT features in reclassified cases revealed that focal or diffuse strong enhancement was found to be the most contributing feature, which led to the correct reclassification of US indeterminate LNs into suspicious LNs, although it led to false positivity in three cases.

Nonetheless, CT is known to be undoubtedly inferior to US with regard to the detection of the preserved fatty hilum, which is required for the probably benign diagnosis of LNs on imaging. This is the reason why many probably benign LNs on US were categorized as indeterminate LNs on CT and why the malignancy risk of the indeterminate category on CT was relatively low and was not different from that of the probably benign category on CT. Moreover, the incidence of additional metastasis found in US probably benign LNs with the addition of CT was very low (1.7%), which means that LNs with probably benign US features are likely to be benign, if properly evaluated, even if there are some indeterminate or suspicious features noted on CT.

Clinical implications of our findings are as follows. First, for the long-standing debate as to whether CT is necessary, this study based on node-by-node analysis supports the rationale for adding CT to standard US examinations. Second, the addition of CT can also help refine candidates for FNA indication among US indeterminate LNs, given that US indeterminate LNs are not infrequently encountered in routine practice, and it may not be practical to perform FNA for all such LNs. The patient-level diagnostic benefit due to increased accuracy may be of great clinical importance at present because active surveillance is emerging as a potential option for patients with papillary thyroid microcarcinoma who do not have LN metastasis [28]. Third, our findings demonstrate that the addition of CT to US may suggest the level of diagnostic confidence for malignancy. Specifically, we can further classify the same US suspicious LNs as suspicious LNs with high diagnostic confidence (concordant suspicious) or suspicious LNs with low diagnostic confidence (discordant suspicious) based on the concordance between US and CT studies. Based on the results, we may skip FNA for concordant suspicious LNs because of the high probability for malignancy and may focus only on discordant LNs in some cases.

Our study had several limitations. First, owing to the retrospective nature of this study, our results were inevitably subject to selection bias. Second, as this study dealt with LNs depicted on US, the results are limited to macroscopic metastatic tumors, excluding microscopic metastatic tumors. However, we believe that our study is clinically relevant because many recent papers have highlighted the prognostic significance of clinically apparent LNs [4,29]. Third, malignancy risks of the US probably benign and indeterminate categories in this study could have been overestimated because not all patients with US probably benign and indeterminate LNs at the initial US evaluation underwent FNA. A future prospective study based on a larger sample size is warranted to validate our findings.

## 4. Materials and Methods

This retrospective study was approved by the institutional review board of our hospital (H-1506-107-682, 29 June 2015), and the requirement for informed consent was waived due to the retrospective nature of the study.

### 4.1. Patient Selection

Patients who had undergone fine-needle aspiration (FNA) or core-needle biopsy (CNB) between Dec 2006 and June 2015 for neck lesions at our institution were selected from our radiology report database. Of the 55,276 patients, 55,051 patients were excluded for the following reasons: (1) a history of head and neck disease or other malignancy (*n* = 6768), (2) a history of previous surgery for thyroid cancer (*n* = 793), (3) FNA or CNB for lesions other than LNs (*n* = 47,427), (4) no corresponding CT imaging (*n* = 59), or (5) non-diagnostic results on biopsy (*n* = 4). Of the 280 LNs identified in the remaining 225 patients, six LNs were excluded because CT and US images could not be correlated in the LNs.

**Figure 6:**
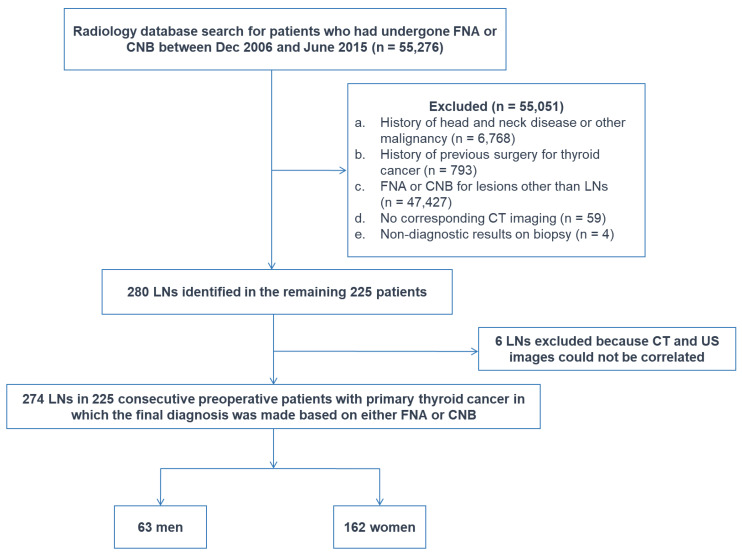
Flowchart of study sample. CNB—core-needle biopsy, FNA—fine-needle aspiration, LN—lymph node.

The final study population included 274 LNs in 225 consecutive preoperative patients with primary thyroid cancer (63 men and 162 women; mean age, 47 years; age range, 18–82 years), in which the final diagnosis was made based on either FNA or CNB (Figure 6). The study population overlaps with that of the previous study which focused on the investigation of malignancy risk and US findings predictive of malignancy for US indeterminate LNs in preoperative thyroid cancer patients [20].

### 4.2. Image Acquisition

All US images were obtained by faculty radiologists, board-certified radiologists who participated in head and neck radiology fellowship training, or residents under faculty supervision using linear transducers (7.5–15 MHz). All CT images were obtained in the cephalocaudal direction from the skull base to the aortopulmonary window with 16–128 channel multidetector CT scanners. Postcontrast CT images were acquired forty seconds after intravenous injection of 90 mL of iodinated non-ionic contrast agent (at a rate of 3 mL/sec) via an automated injector, followed by 20–30 mL of normal saline for flushing, with collimation of 0.625–1.25 mm and a pitch of 0.9–1.3. In some patients, additional precontrast CT images were obtained for better depiction of calcifications. All CT images were reconstructed into axial images and, in part, coronal images for review at every 2.5–3 mm on a 512 × 512 matrix. The median time interval between US and CT examinations was 19 days (interquartile range, 9–40 days).

### 4.3. Image Analysis

All US and CT images were independently analyzed by two thyroid radiologists (R.E.Y. and J.H.K. with 8 and 17 years of experience performing thyroid US imaging, respectively) and cases with disagreement were reviewed together by the two reviewers at a separate session to reach a consensus. On US, LNs were first assessed in terms of: (1) location: LN level and compartment (central [level 6] vs. lateral [level 1 to 5]) according to the nodal classification scheme of the American Joint Committee on Cancer level system [6,30], (2) size of the primary tumor (largest diameter), and (3) sizes of LNs in the most representative longitudinal nodal plane (short diameter [SD] and long diameter [LD]).

US images at the most representative slices of the LNs were analyzed for the following characteristics: (1) echogenicity relative to the anterior neck muscles (the strap or sternocleidomastoid muscles) (hyperechoic [diffuse or focal], isoechoic, or hypoechoic), (2) presence or absence of an echogenic hilum, (3) presence or absence of calcification, (4) presence or absence of cystic change, and (5) vascular pattern on color Doppler images (none, hilar pattern, or peripheral or diffuse) [10]. Subsequently, CT images were correlated with US images on a node-by-node basis to identify the LNs depicted on US. On CT, the LNs were analyzed for imaging features, including the contrast enhancement pattern and the presence or absence of a fatty hilum as well as cystic change or calcification. The contrast enhancement pattern was classified into four patterns: (1) strong enhancement (focal or diffuse), (2) heterogeneous enhancement, (3) hilar vessel enhancement, and (4) others [10].

For the LN classification, LNs were categorized as probably benign (or normal), indeterminate, or suspicious LNs based on each modality [8,10,20]. Probably, benign LNs were defined as those with either an echogenic hilum or hilar vascularity on US and those with either central hilar fat or central hilar vessel enhancement on CT in the absence of any suspicious features [10]. LNs were interpreted as suspicious if any one of the following features was present on US or CT: (1) calcification on US or CT, (2) cystic change on US or CT, (3) hyperechogenicity compared to the adjacent muscles on gray scale US, (4) peripheral or diffuse color Doppler pattern, (5) focal or diffuse strong enhancement on CT, or (6) heterogeneous enhancement on CT. Indeterminate LNs referred to LNs with no imaging features of suspicious or probably benign LNs and included those with neither a hilar fat nor hilar vascularity on US or CT regardless of the nodal shape [10]. In the combined analysis of US and CT, LNs with any suspicious features on either of the two modalities were categorized as suspicious LNs. On the other hand, indeterminate LNs with probably benign or suspicious features on either of the modalities were categorized as probably benign or suspicious LNs, respectively.

### 4.4. Statistical Analysis

All statistical analyses were performed using the statistical software MedCalc, version 11.1.1.0 (MedCalc, Mariakerke, Belgium). The Kolmogorov–Smirnov test was used to assess the normality of numerical data. In all tests, *p*-values less than 0.05 were considered statistically significant.

Incidences and malignancy risks were calculated for each diagnostic category on US, CT, and combined US/CT and were compared using Fisher’s exact test. The concordance rate between US and CT categories was also calculated, and the malignancy risks of concordant and discordant cases were compared using Fisher’s exact test. The word ‘concordant’ was used when categories were the same between US and CT. ‘Discordant suspicious’ refers to cases classified as the suspicious category on either CT or US and as the probably benign or indeterminate category on the other imaging modality. ‘Discordant benign’ refers to cases classified as the probably benign category on either CT or US and as the indeterminate category on the other imaging modality. Interobserver agreement for the US and CT classification of LNs was assessed using Cohen’s unweighted kappa (κ) coefficients. A κ coefficient of 0.00–0.20 was considered to indicate slight agreement, 0.21–0.40 fair agreement, 0.41–0.60 moderate agreement, 0.61–0.80 substantial agreement, and 0.81–1.00 almost perfect agreement [31].

## 5. Conclusions

In conclusion, for LN characterization in patients with thyroid cancer, the addition of CT to US has the potential to be of value by suggesting the level of diagnostic confidence for the suspicious category according to the concordance of US and CT studies and by helping correctly detect metastasis in US indeterminate LNs.

## Figures and Tables

**Table 1:** Incidences of the US, CT, and combined US/CT categories.

Category	US	CT	Combined US/CT
Probably benign	21.2 (58/274)	8.4 (23/274)	20.4 (56/274)
Indeterminate	24.5 (67/274)	36.9 (101/274)	18.2 (50/274)
Suspicious	54.4 (149/274)	54.7 (150/274)	61.3 (168/274)

Data are percentages (raw data). CT—computed tomography, US—ultrasonography.

**Table 2:** Malignancy risks of the US, CT, and combined US/CT categories.

Category	US	CT	Combined US/CT
Probably benign	1.7 (1/58)	8.7 (2/23)	0 (0/56)
Indeterminate	22.4 (15/67)	5.9 (6/101)	8.0 (4/50)
Suspicious	77.2 (115/149)	82.0 (123/150)	75.6 (127/168)

Data are percentages (raw data). CT—computed tomography, US—ultrasonography.

**Table 3:** Malignancy risks of the combined US/CT categories according to the US and CT categories. Unless otherwise indicated, data in parentheses are the number of LNs.

US	CT	Combined US/CT	Malignancy Risk % *
	Probably benign (16)	Probably benign (16)	0 (0/16)
Probably benign (58)	Indeterminate (38)	Probably benign (38)	0 (0/38)
	Suspicious (4)	Suspicious (4)	25.0 (1/4)
	Probably benign (2)	Probably benign (2)	0 (0/2)
Indeterminate (67)	Indeterminate (50)	Indeterminate (50)	8.0 (4/50)
	Suspicious (15)	Suspicious (15)	73.3 (11/15)
	Probably benign (5)	Suspicious (5)	40.0 (2/5)
Suspicious (149)	Indeterminate (13)	Suspicious (13)	15.4 (2/13)
	Suspicious (131)	Suspicious (131)	84.7 (111/131)

* Data in the parentheses are raw data. CT—computed tomography, US—ultrasonography.

**Table 4:** Concordance between the US and CT categories.

US/CT Category *	No. of LNs (%)	Malignancy Risk % ^†^
Concordant benign	16 (5.8)	0 (0/16)
Discordant benign	40 (14.6)	0 (0/40)
Concordant indeterminate	50 (18.2)	8.0 (4/50)
Concordant suspicious	131 (47.8)	84.7 (111/131)
Discordant suspicious	37 (13.5)	43.2 (16/37)

* The word ‘concordant’ was used when categories were the same between US and CT. ‘Discordant suspicious’ refers to cases classified as the suspicious category on either CT or US and as the benign or indeterminate category on the other imaging modality. ‘Discordant benign’ refers to cases classified as the benign category on either CT or US and as the indeterminate category on the other imaging modality. ^†^ Data in the parentheses are raw data. CT—computed tomography, US—ultrasonography.
